# Landslide Susceptibility Assessment Using Spatial Multi-Criteria Evaluation Model in Rwanda

**DOI:** 10.3390/ijerph15020243

**Published:** 2018-01-31

**Authors:** Jean Baptiste Nsengiyumva, Geping Luo, Lamek Nahayo, Xiaotao Huang, Peng Cai

**Affiliations:** 1 State Key Laboratory of Desert and Oasis Ecology, Xinjiang Institute of Ecology and Geography, Chinese Academy of Sciences, No. 818, South Beijing Road, Urumqi 830011, China; jbatigol@yahoo.com (J.B.N.); lameknahayo@gmail.com (L.N.); hxt1983@gmail.com (X.H.); caipeng13@mails.ucas.ac.cn (P.C.); 2 University of Chinese Academy of Sciences, Beijing 100049, China; 3 Ministry of Disaster Management and Refugees (MIDIMAR), P.O. Box 4386, Kigali 00250, Rwanda

**Keywords:** disaster, GIS, hazard, landslide, Rwanda, Spatial Multi-Criteria, susceptibility

## Abstract

Landslides susceptibility assessment has to be conducted to identify prone areas and guide risk management. Landslides in Rwanda are very deadly disasters. The current research aimed to conduct landslide susceptibility assessment by applying Spatial Multi-Criteria Evaluation Model with eight layers of causal factors including: slope, distance to roads, lithology, precipitation, soil texture, soil depth, altitude and land cover. In total, 980 past landslide locations were mapped. The relationship between landslide factors and inventory map was calculated using the Spatial Multi-Criteria Evaluation. The results revealed that susceptibility is spatially distributed countrywide with 42.3% of the region classified from moderate to very high susceptibility, and this is inhabited by 49.3% of the total population. In addition, Provinces with high to very high susceptibility are West, North and South (40.4%, 22.8% and 21.5%, respectively). Subsequently, the Eastern Province becomes the peak under low susceptibility category (87.8%) with no very high susceptibility (0%). Based on these findings, the employed model produced accurate and reliable outcome in terms of susceptibility, since 49.5% of past landslides fell within the very high susceptibility category, which confirms the model’s performance. The outcomes of this study will be useful for future initiatives related to landslide risk reduction and management.

## 1. Introduction

Landslides are confirmed severe forms of natural disasters [1] and most of them are caused by specific geological, geomorphological and climatological conditions as well as anthropogenic activities [2]. Landslides have been classified as the third most dangerous disaster [3], since they cause huge fatalities as well as enormous damages, especially in hilly topographic zones globally [4]. It is therefore necessary that strong and adequate measures are provided for preventing landslides and mass movements which will contribute to reducing associated impacts [5]. In many cases, this is not easily feasible due to various reasons, thus innovative and realistic approaches have to be adopted for enhancing landslides’ risks management, and their susceptibility must be well mapped to enable rational decisions in line with landslide risk management [6,7].

In fact, landslide disasters have serious and diverse impacts. Globally, existing figures have confirmed their rise in damages and losses. The figures are much more serious in the last decade, with 32,322 fatalities recorded; monitoring, mapping and forecasting of these landslide hazards are less than adequate as required within different countries in the world [8]. Fast moving landslides such as the rockslides, debris flows, rock falls, avalanches and others generated huge number of deaths and related consequences, and continue to increase, particularly in mountainous regions [9]. For some places, anthropogenic factors have increased landslides with highly recurrent rainfalls, and a great number of today’s mass movements are related to the past landslide events [10]. According to the United Nations’ analysis [11], 346 disaster incidents occurred in 2015 and killed around 22,773 people. From the same disasters, 98.6 million people were affected, while USD66.5 billion were economically lost and, among these disasters, landslides alone affected 150,332 in 2015 and killed 4369 worldwide.

Landslides in Rwanda, similar to most of East Africa, are among the very deadly natural disasters that are taking lives and damaging enormous properties. Rwanda loses more fertile soils to foreign water catchments within the Nile and Congo river catchments, where 15,000,000 tons are lost on average every year [12]. Annual high intense rainfalls particularly affect the soils negatively by providing high saturation of soil profiles, resulting in frequent occurrences of landslides [13,14]. In addition, high population pressure to land also results in the Environmental disasters and encroachment on the fragile ecosystems [15]. Hence, Rwanda’s topographic nature is entirely hilly, and the country’s steep slopes areas are extremely prone to landslides [14,16]. Moreover, as previously reported [12,17], Rwanda has a high density of landslides with a shortage of related scientific research, making it an excellent site for study using modeling techniques for prediction, therefore bridging scientific gaps of landslide studies in Rwanda. Although it is clear that landslides cause enormous damages in different parts of Rwanda, the causal factors that really influence them are still not well understood, and their susceptibility and critical high prone zones are still unidentified. The reason some hilly areas are not as affected as others that are heavily devastated leaves more critical research questions to be answered by scientific approaches.

Available data indicate that heavy rains experienced during October–March every year cause landslides [18], which have affected around 8000 people from 1963 to 2010, among them 45 died and many houses were destroyed [19]. Subsequent to the creation of a nodal institution in charge of coordinating disaster management activities (MIDIMAR), the systematic disaster recording system was installed, and from 2013 to 2016, 174 deaths, 122 injuries, more than 5000 house collapses, and many hectares of crops being washed away by landslide disasters were recorded. Such condition stresses the rationale to conduct a detailed landslide susceptibility study for Rwanda in order to overcome all these losses and curb the impacts [4]. Efforts, therefore, must be in highlighting unstable zones by using suitable methodologies since it can provide sustainable response and protect lives and properties [1]. In this perspective, landslide susceptibility study can deliver valuable information useful for hazard mitigation through appropriate planning [20].

Thus far, no attempt has been made to predict these landslides and prevent damages caused by them in the study area using suitable scientific tools. Only few recent disaster management studies were conducted in Rwanda with main focus on hazard description, risk and vulnerability analysis, awareness and capacity building, early alert and warning. All these were done by applying descriptive, secondary data sources and social approaches limited to the district levels and also without focusing on one single hazard by considering as many causal factors as possible [12,15,17,21], and, as confirmed by Westen [22], any hazard mapping and study activity related to disaster risk has to be preceded by susceptibility assessment. However, a landslide susceptibility assessment of hilly regions such as Rwanda has to be thoroughly conducted using appropriate methodologies to anticipate all associated impacts, subsequently serving as a baseline for other studies. In doing so, it is important to consider several causal factors that influence landslide while using important models to map landslide susceptibility in Rwanda.

For large-scale landslide susceptibility studies, a range of methodologies have been proposed [23,24], but very few limited studies have been conducted on landslide susceptibility assessment for large areas and entire countries [9,25]. In studying landslide susceptibility, causal factors and indicators are more important in producing susceptibility and risk maps. The number of factors to consider varies from one study to another due to its objectives, available data, time availability, landslide types, historical records, country situation, level of the landslide hazard and others. Thus far, wide ranging literature has emphasized on the factors that may be considered when studying landslide susceptibility.

For the landslide study of Cuba using Spatial Multi-Criteria Evaluation, Castellanos et al. [26] considered ten factors: Slope angle, land use, geology, rainfall, geomorphology, slope length, drainage density and internal relief, precipitation and seismicity. Therefore, the Food and Agriculture Organization of the United Nations (FAO) has proposed some causal factors that might be considered in the study of erosion and landslides such as soils, geology, rainfall, weathering, topography, vegetation and land use, groundwater as well as human activities [27]. At this stage, the selection of factors depends on the study aim and data availability. Additionally, different researchers have used various causal factors while studying landslide susceptibility, Bayes [28] considered nine factors (distance to streams, distance to roads, precipitation, distance to drain, Normalized Difference Vegetation Index (NDVI), land cover, elevation, slope and soil permeability) for the susceptibility mapping of Bangladesh with Spatial Multi-Criteria Evaluation (SMCE). Therefore, it is clear that, for any study of landslide susceptibility, several causal factors are applied to generate accurate susceptibility maps. Besides, other studies considered different causal factors for landslide susceptibility mapping: Pourghasemi et al. [29] considered slope degree, slope aspect, altitude, plan curvature, profile curvature, surface area ratio and topographic position index, while Pradhan et al. [24] referred to nine factors, namely distance to fault, lithology, distance to roads, land use, slope degree, aspect, elevation, distance to rivers and curvature for the study of landslide susceptibility of Iran by applying SMCE. The above landslide causal factors are categorized into four groups: geological, anthropogenic, hydrological and geomorphological. In Rwanda, Piller et al. [15] studied landslide using logistic regression with some environmental factors such as slope, soil type, land cover and precipitation. They related precipitation with landslide and found that precipitation is an important cause of landslides in Rwanda.

It is mandatory to provide a clear picture of potential occurrence and this has to be done by considering the causal factors that led to the past slope failure to inform on the current study on susceptibility [22,30]. For the landslide susceptibility assessment in Rwanda, eight causal factors have been taken into account: slope, distance to roads, lithology, precipitation, soil texture, soil depth, altitude and land cover. These were selected based on the objectives of the current study, information from the fieldwork, historical records and available data. Therefore, the objectives of this study are to: (1) generate landslide susceptibility maps; (2) highlight the most vulnerable zones and/or safer zones to landslides; and (3) divulge population exposure to landslide disasters using Spatial Multi-Criteria Evaluation Model in Integrated Land and Water Information System (ILWIS) of Geographic Information System.

## 2. Data, Materials and Methods

### 2.1. Study Area Profile

**Figure 1:**
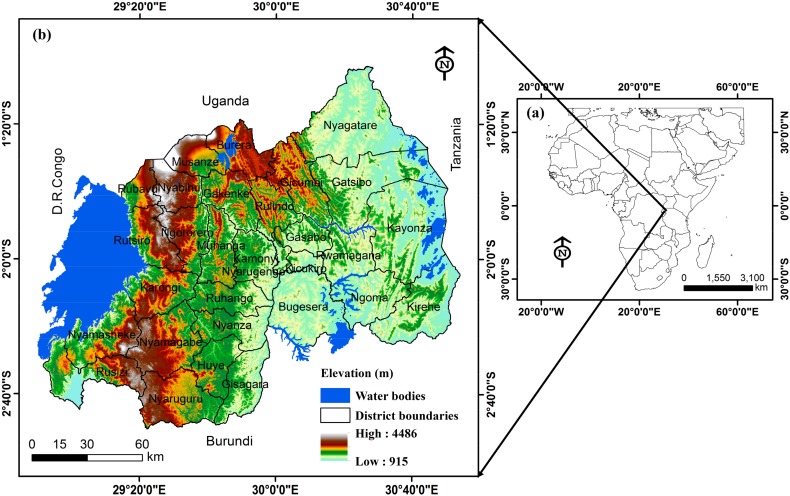
Location map of Rwanda: (**a**) a map of Africa for Rwanda localization; (**b**) a map of Rwanda with 30 districts and elevation in meters.

This current research covered the entire Rwanda, an African country situated in the east (Figure 1). Rwanda is composed by 30 districts under four Provinces and the City of Kigali, with a total surface area of 26,338 square kilometers. This land-locked territory lies in the east of the surroundings of the Kivu Lake, and has a total population of 11,809,295 in 2016. It is geographically bound by 1–3° S latitude, 28–31° E longitude [31].

Rwanda is surrounded in the north by Uganda, in the south Burundi, the Democratic Republic of Congo (DRC) in the west and in the east by the United Republic of Tanzania. Rwanda extends over an unstable mountainous area and its topography is generally characterized by steep slopes [18,32].

Generally, high and steep mountains up to 4486 meters above sea level dominate northwest and central parts of the study area. This mountainous topographic nature is mostly dominant in the western and the northern parts of Rwanda. The highest peaks are found in the Virunga Volcano chain in the northwest including mount karisimbi, Rwanda’s highest point [33]. Landslide hazards are very common phenomena in Rwanda, due to its topographic nature associated with other causal factors. Most of these landslides frequently occur in almost all the districts composing the study area and cause many shocks.

These figures were adapted from the Ministry of Disaster Management and Refugees in Rwanda (MIDIMAR), and Field surveys conducted by the researchers (January–September 2017). They highlight some major landslide events and associated impacts and effects in different parts of the country within different periods of time to confirm the severity of landslide hazards and disasters in the study area.

The above data highlight some of the major events caused by landslide hazards in recent years with a web of impacts in different parts of the country (Table 1). Landslides usually occur from March to May for several reasons including high precipitation [18] and other different causal factors. This has become a frequent devastating phenomenon which needs serious and particular attention to restrain all related impacts that overwhelm the people living in prone zones and undermine development initiatives.

**Figure 2:**
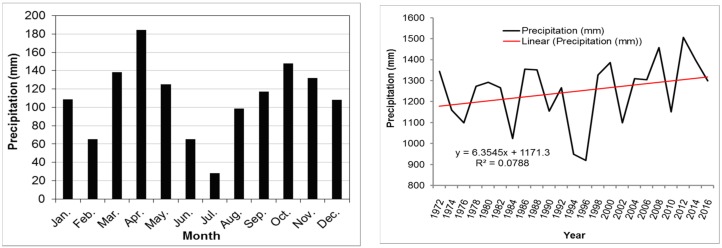
Monthly precipitation pattern and annual precipitation dynamics of Rwanda country from 1972 to 2016.

For Rwanda Country, it was confirmed that the precipitation aspects of the country vary largely in space and in time (Figure 2) and this is caused by many different factors including its geo-spatial localization. Rwanda has two main rain seasons: one in the beginning (March–May) of the year and another one towards the end of the year (October–December). Due to these continuous changes in rainfall patterns, an increase of serious weather related hazards including landslides that impact the country at different scale, scattered nationwide is registered [19]. This confirms the rationale for landslide susceptibility mapping. Additionally, the country has a double weather foundation explained by the phenomenon of the sun that crosses the equator around March, and the southern summer around September each year.

### 2.2. Datasets

#### 2.2.1. Landslides Inventory Map

As previously reported [24,28,34,35], every susceptibility assessment research must be based on a factual collection of past records of events to serve as the basis and to guide the entire process. Within Rwanda, 980 past slide locations have been identified and collected by referring to secondary data sources from existing documents such as old maps and photographs, reports from Ministry of Disaster Management, disaster databases, disaster loss inventory (DesInventar), disaster reporting and monitoring system in Rwanda together with extensive field data collection from March to October 2017 on landslide hazards using GPS and with the help of local residents. This was done as an entry point for modelling landslides susceptibility in Rwanda and no other previous research work has produced landslide inventory mapping.

**Figure 3:**
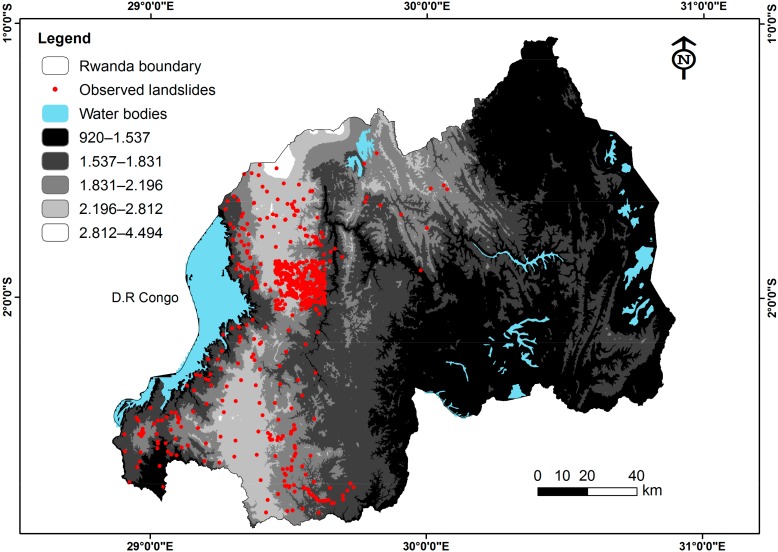
Rwanda observed landslides inventory map.

Some major areas are affected by past landslides, as identified while conducting field visits (Figure 3); by using Global Positioning Systems (GPS), and secondary information sources obtained at the Ministry of Disaster Management and Refugees (MIDIMAR), Lands and Mapping Agencies and District Land Offices as well. In addition, field investigations and modelling studies can enhance the knowledge on slope movement and provide strong solutions to landslides related problems [36].

#### 2.2.2. Landslide-Related Causal Factors

To study the landslide susceptibility, the researcher must be able to recognize the real causal factors that might lead to instability in a given area. This information is, therefore, key, since it helps in achieving accurate findings upon completion [6]. For the current study, eight landslides related causal factors were selected: distance to roads, slope, lithology, precipitation, altitude, soil depth, soil texture and land cover/use. The semi-quantitative approach considers explicitly several factors influencing the slope stability [37]. For deducing these eight causal factors, researchers consulted the Rwanda national disaster risk management plan [14], the national disaster management policy [38] and the national contingency plan for floods and landslides [21]. Additionally, field visits were conducted in all 30 districts of Rwanda to pinpoint locations where landslides occurred in past, and to identify the potential causes that might have caused the instability of the slope. The previous study on landslide hazard in Rwanda by Piller [15] also inspired the researchers on deducing eight causal factors. The SMCE, as an advanced model, incorporates an expert based knowledge approach, essential in making the selection of relevant factors for the susceptibility assessment [39].

To develop the map the study area, researchers used the digital elevation model (DEM) of Rwanda with the help of the slope function in ArcMap 10.3 (Redlands, CA, USA), and, thus, the slope map was derived. The slope map was categorized into four classes to help in landslide susceptibility assessment [40]. The DEM (30 m resolution) was obtained from the United States Geological Survey (USGS) [41]. ArcMap 10.3 (Redlands, CA, USA) was also used to derive the altitude of the study area from the DEM which is another landslide causal related factor.

To consider land cover/use factor in landslide susceptibility assessment, the Rwanda updated land cover map of 2016 was classified with data obtained from Landsat-8 images delivered by the USGS [41,42], appropriate remote sensing and GIS software, together with adequate techniques in Envi 5.3 and ILWIS Software packages [43,44].

**Figure 4:**
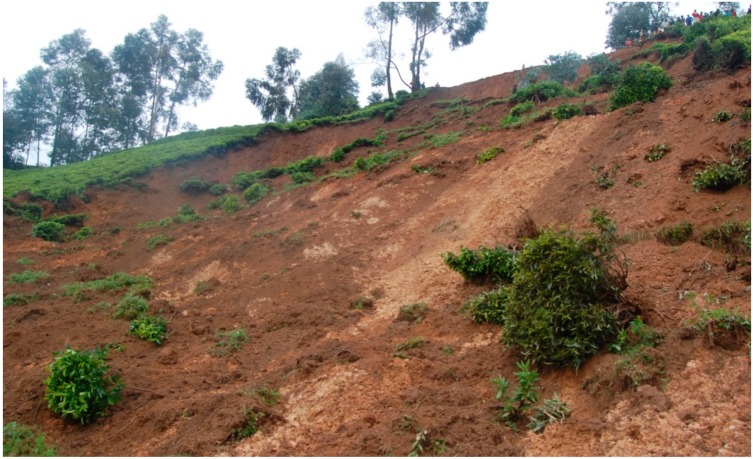
Areas affected by landslides in the study area (field visits to Rwanda, January–September 2017).

The illustrations in Figure 4 confirm the presence of landslide events in the study area, where they devastate different areas and damage a wide range of properties including houses, roads, bridges, infrastructure facilities, crops and environment and, in most cases, families are left homeless.

**Figure 5:**
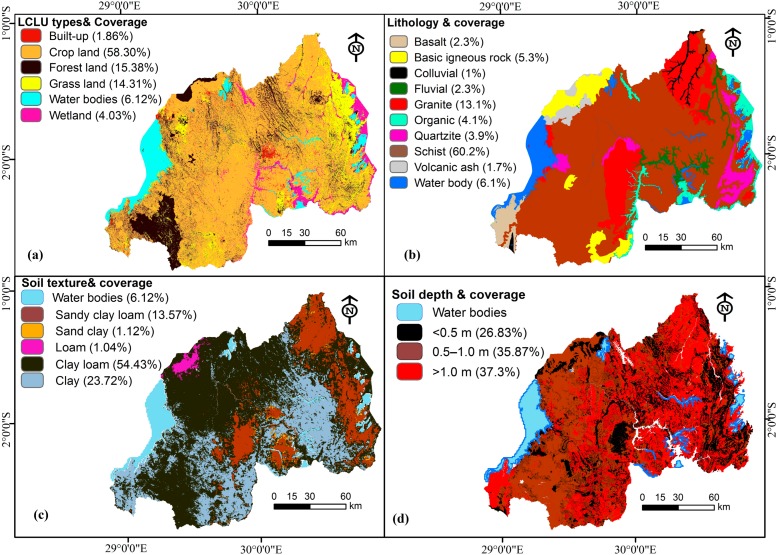
Landslide conditioning factors in the study area: (**a**) Land cover land use types, (**b**) Lithology, (**c**) Soil texture, (**d**) Soil depth.

The land cover/land use map was then classified following the previous East-African classification done by the Regional entre for Mapping of Resources for Development (RCMRD) [45] for land use maps of Rwanda. The information from the latter guided the land cover/use classification in this study. Additionally, the USGS classification method, type one was also applied [43,46]. Rwanda was hence classified into six land cover/use types (Figure 5a) and it is dominantly covered by cropland (58.30%) followed by forest land (15.38%).

Moreover, geological and lithological features of the study area were derived from available mining and geological maps of Rwanda in good scale (1:100,000) [47], soil map database from the national soil surveys, and mapping of the study area by the Rwanda ministry of agriculture in 1995 [48,49,50]. These datasets provided landslide causal factors including lithology, soil texture and soil depth under different classes (Figure 5 and Table 2).

**Figure 6:**
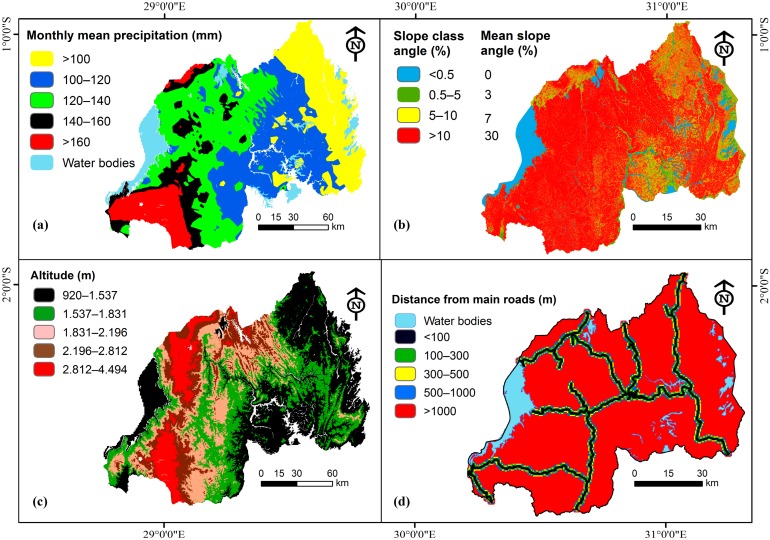
Landslides conditioning factors: (**a**) monthly mean precipitation, (**b**) slope, (**c**) altitude and (**d**) distance to main roads.

It has been confirmed by previous scientific studies that, for regions with hilly topographic nature, disturbances such as construction of new roads, excavation activities and other different human-made activities may cause landslide hazards [51,52], and, therefore, researchers judged reasonable to take into account the distances from main roads as a landslide causal factor while conducting susceptibility assessment studies. The delineation of the distance from the main roads in Rwanda was done by creating Euclidean distance from 100 m in ArcMap-Spatial Analyst extension (Figure 6), and this was completed using the road network datasets of the study area obtained from the Rwanda Ministry of Infrastructure under Transport and Development Agency (RTDA) [53].

For long-term annual and monthly mean precipitation intensity, this research utilized monthly and annual rainfall mean for 44 years (1972–2016). The datasets were obtained from Frank, et al. [54] and these contain information from 1981 to 2016. These data were used in conjunction with datasets obtained from the Rwanda Meteorological Agency form all meteorological stations nationwide [55] from 1972 to 2016 (Figure 2 and Figure 6). The combination of both datasets provided complete, sufficient and reasonable information to help researchers run the model appropriately and produced landslide susceptibility maps.

To assess the population exposure to landslides in Rwanda, population datasets were obtained from the Rwanda National Institute of Statistics [56,57] and the free online datasets from Statoids sources for countries [58]. All above datasets have been organized and standardized following the appropriate methodologies described to generate the landslide susceptibility maps. Many studies on landslides take into account the slope orientation as a landslide causal factor [10,24,59]. Conversely, the orientation of slope, which is considered as a landslide causal factor in many susceptibility studies across the world, was not taken in account for the present study, as it never influences soils temperature parameters, especially within tropical zones where Rwanda is also located [15]. Thus, the slope aspect was not considered as a landslide causal factor in the analysis of landslide susceptibility in Rwanda.

Landslide conditioning factors are important to take into consideration in assessment of natural hazards and clear knowledge and information about the major landslide-related factors is required to detect the susceptible and prone zones. Consequently, it was decided to start by generating an adequate data catalogue for present study to visualize the extent of landslide, types of historical landslide events and identify potential causes as well. In the same line, various studies confirm that this is a pre-requisite in any landslide modeling activity [28,60,61,62].

This study followed this principle and eight causal factors have been considered including: land cover, lithology, soil texture, soil depth, precipitation, slope, altitude; and distance to main roads. These causal factors have been used to model the landside susceptibility of Rwanda to highlight areas that are likely to be prone to landslides at any given moment of time. In the principles of disaster risk management, susceptibility mapping is key, since it guides all planning and decision making process as far as landslides mitigation is concerned.

As indicated above, eight causal factors were considered in studying landslide susceptibility of Rwanda (Figure 5 and Figure 6).

#### 2.2.3. Methodology

**Figure 7:**
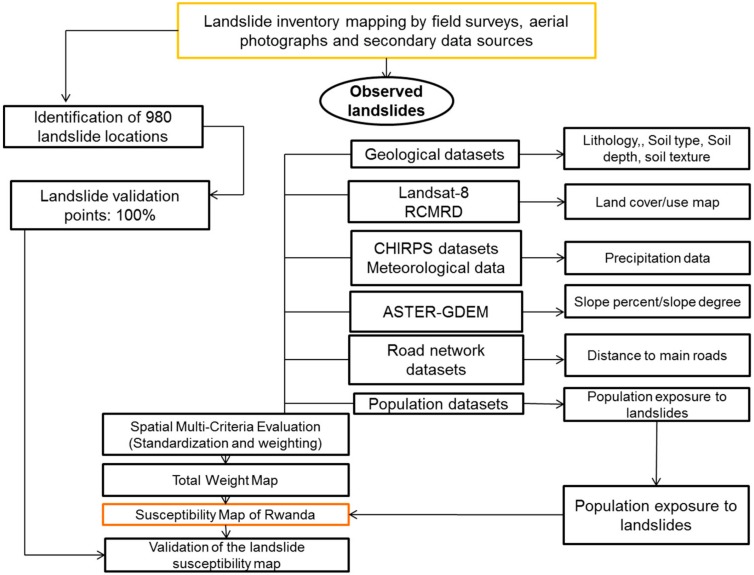
Flowchart of methodology.

The current study aimed to conduct landslide susceptibility assessment in Rwanda, as stated above, by using the semi-quantitative approach (Spatial Multi-Criteria Evaluation Model). The multi-criteria evaluation approach was adopted by this study due to its advantages of combining both qualitative and quantitative information. It includes an expert based knowledge approach [64], and this leads to producing accurate landslides susceptibility maps at a national scale. The methodology flowchart employed in the current study is illustrated in Figure 7. Given data shortage, time limitations, objectives of the study, and Rwandan spatial features, researchers opted to apply semi-quantitative modeling approach. The hazard assessment and mapping can therefore utilize existing and consistent datasets [29,59,65]. This methodology has been ascertained to be suitable for some circumstances, especially: (a) when landslide susceptibility assessment is scratching from the ground, as starting stage; and (b) when acquiring enough and sufficient datasets for a national scale is challenged by some limitations such as lack of numerical datasets, absence of a complete landslide inventory covering the entire study area, inaccessibility to some prone areas and time constraints. In addition, the available DEM for Rwanda (10 m resolution) was not complete for some areas bordering the Democratic Republic of Congo. Thus, researchers have generated a digital elevation of 30 m resolution in the current study. The SMCE model considers a range of landslide related causal factors and for this study only eight were considered (Figure 5 and Figure 6). These factors have been prepared for the production of susceptibility maps of Rwanda.

The application of this methodology requires grouping, standardizing, scoring and weighting all input parameters under the format of maps [26] to determine the contribution of each in producing landslide hazards and disasters in Rwanda. These factors do not contribute equally in causing a landslide event. SMCE is embedded within the theoretical framework initiated by Saaty [24] for spatial analysis and susceptibility modeling. Thus, the application SMCE model is made possible by standardization of original inputs data parameters and layers to 0–1 value range and to handle this transformation, specific methodologies and equations may be applicable. ILWIS Module for SMCE provides different standardization methods. This facilitates the compatibility of model with input datasets and allows the user to produce the most reliable susceptibility map. The quality of the output maps depends on quality and accuracy of the used inputs, as well as the applied techniques.

The scoring and weighting were done following the studies of Abella et al. [26] and Westen [23], and, to implement this, the Spatial Multi-Criteria Evaluation module of ILWIS-GIS was followed. Once the researchers have classified the input datasets to use for the susceptibility assessments, weighting is done relating to the contribution level of each factor in causing the past landslide occurrences within a specific area. The weight of a causal factor equals its influence on the overall objective. Field observations in the study area were conducted to identify areas prone to landslides. This was done from interviews with local community members, indigenous knowledge holders, historical records and direct observations. From this, possible causes of slope instability were identified.

The assumptions highlight that the landslide occurrence is caused by the different contribution of causal factors. The scoring of causal factors also follows that principle. In principle, factors do not contribute equally at any landslide event and weights are assigned differently from 0 to 1 as above mentioned. The SMCE model in Integrated Land and Water Information System of GIS was used to make all necessary weighting and other required computations to produce Rwanda landslide susceptibility maps.

Moreover, there exist various ways of presenting the spatial decision making process [65]. Alternatives can be used in a matrix format X with several criteria (y_1_ to y_n_). The decisions are then made in accordance with a list of possible alternatives. From that procedure, cells are presented for alternative performance in producing susceptibility (X_ij_) in terms of landslides hazard modeling. Every cell’s value is obtained by multiplying standardized input (0–1) and the weighted one. The total cell value gives the final value for a particular criterion within the given alternative.

(1) $$x_{\mathrm{ij}}=V_{\mathrm{ij}}^{*}\overset{h}{\underset{L=0}{\prod }}W_{j}^{L}$$

where X_ij_ is the standardization value of criterion (Y_j_) for (X_i_) alternative, and $w_{j}^{L}$ is the weight of a criteria for levels 0–h in the modeling of the landslide.

For the assessment process, it would be advisable to assign a standardized range to continuous factors and thereafter combine weighted average. A susceptibility map is therefore obtained from the combination of all considered factors’ weights.

**Figure 8:**
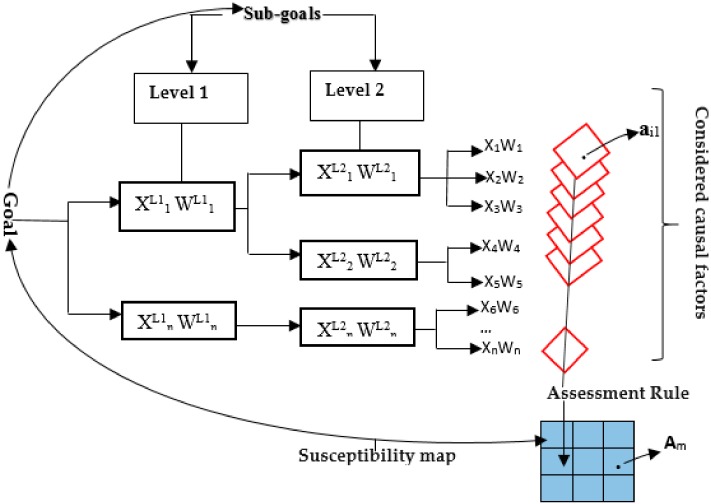
Spatial Multi-Criteria Evaluation Schematic illustration. X, criterion; W, Weight; L, level; A, Alternative; M, number of alternatives; N, number of criteria; a_ij_, Performance of alternative i and criterion j.

For the current study, the weighting of causal factors was done by SMCE_ILWIS. For implementing this, the above matrix (Table 3) was used within ILWIS Environment, by employing the ranking of the criteria. The synthesis is therefore related to the multiplication among the hierarchical process. In addition, when implementing this, every criteria (Y_j_) becomes a raster layer and every pixel or set of pixels of the final map becomes eventually an alternative (X_j_). From this principle, the generation of weights for causal factors in the current study is well illustrated in Figure 8, where they have been computed by the SMCE model. The goal has been decomposed in criteria levels. Generally, criteria consist of raster maps and their spatial performance (a_ij_) and alternative (A_j_) are identified for a particular raster cell. The final susceptibility map is therefore obtained by an assessment or decision rule which was calculated following Equations (1) and (2).

Figure 8 illustrates the Spatial Multi-Criteria model (SMCE) as applied in the current study, by the use of a criteria-tree in ILWIS, as modified form Abella [26]. For SMCE model in ILWIS, all indicators and sub-goals are only added. While implementing the model, the actual calculation of weights is run by the model.

(2) $$\mathrm{Si}=\sum^{\text{​}}W_{j}X_{j}$$

where Si is the suitability index, W_j_ is factor j’s weight, and X_j_ is factor j’s Criterion Score. This was applied in the current study to come up with landslide susceptibility maps of the study area.

For the determination of weights, Spatial Multi-Criteria Evaluation model uses three different methods: direct weights, pairwise comparison and rank ordering. In the current study, rank ordering was applied. For this weighting technique, the researcher selects the relative ranking of the factors, and the software calculates and converts these in quantitative weights. Table 4 presents the weights as generated and assigned to causal factors by SMCE model. Weights are always numbers between 0 and 1, and these can never be negative.

As shown in Table 4, the model generated weights for all considered causal factors in relation with their contribution to the occurrence of the past observed landslides. Some factors were ranked less in comparison to others. For example, soil depth was not given much weight in the model, since all past landslides were concentrated in an area with moderate soil depth, while no landslide was observed in the eastern part, which is dominated by a soil depth of more than one meter (Figure 6d). The output is one landslide susceptibility map at national level, with a good scale. The final map indicates the performance of the implemented model.

## 3. Results

**Figure 9:**
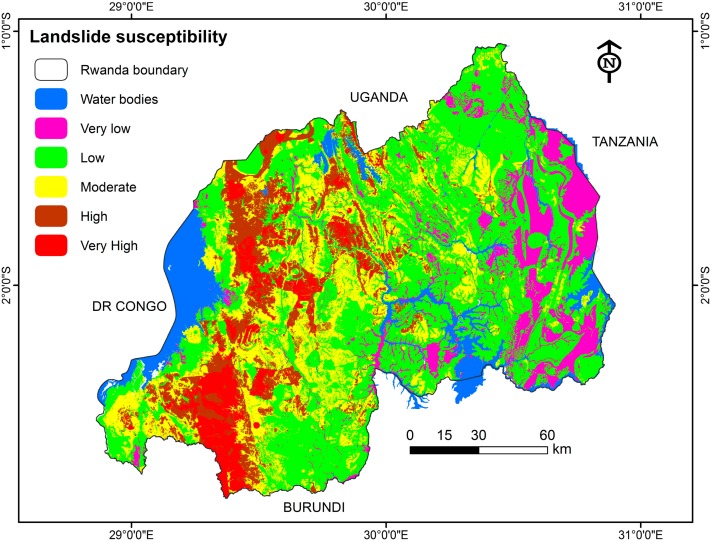
Landslides susceptibility map of Rwanda produced by Spatial Multi-Criteria Evaluation.

To make the Spatial Multi-Criteria Evaluation (SMCE) model more effective in modeling landslide susceptibility, researchers need to combine it with landslide inventory data from the study area [26,64]. This can give a clear understanding of the real causal factors for the area under investigation. For mapping landslide susceptibility in Rwanda, eight related factors (land cover, lithology, soil texture, soil depth, precipitation, slope, altitude, and distance to roads) have been considered. These have produced results on how susceptibility is distributed across the entire territory of Rwanda (Figure 9 and Table 4). Causal factor weighting is of great importance to handle such task and achieve the planned goal of susceptibility mapping through SMCE Modeling. It was thoroughly revealed that the Congo Nile ridge region (Western part of the country) has highest level of landslide susceptibility (40.47%), followed by the North (22.8%), whereas the eastern Province has the lowest (0.85%), as supported by historical landslide records (Figure 1). This also conforms with the landslide effects incurred in recent years (Table 1), where different locations from the west have experienced serious major landslide casualties.

Furthermore, Figure 9 shows that the significant classification of increase of landslide susceptibility in all provinces can be inferred. The increasing landslide susceptibility is ranging from the western to the eastern part. There is an appealing scenario, however, concerning the north part which is also dominated by high susceptibility due to its topographic, geological and morphological nature. Since the area is hilly, the impact of rains to trigger landslide will be easy, resulting in hydrological factor being among the landslide causal factors within the northern area of Rwanda.

Historical landslide events assist in attempting to explain this phenomenon. Highlighting areas exposed and susceptible locations becomes a useful driver to inform decision makers [61]; again, the produced map (Figure 9) presents a clear picture at the national level, which will serve as a baseline in terms of landslide risk management and landslide mitigation in order to minimize the impacts and effects mainly by saving lives and protecting properties from the natural landslide hazards in Rwanda. Recently, the government is struggling to find sustainable solutions to natural hazard risks and impact minimization, as exacerbated by climate change, and this can be considered as one of the possible solutions to address the problem.

From the generated Rwanda landslide susceptibility map (Figure 9 and Table 5), the country presents two facets: landslide high zone (western part) and landslide stable zone (eastern part). All this can be justifiable by different reasons, especially the physical spatial features of the area, and the presence of causal related factors. Due to its hilly topography, the mountainous regions of the country are more likely to be affected by landslides than other areas, and this can be associated by different huge losses caused by landslides. Considering the areas under landslide categories through generated maps, the study area is confirmed to be highly susceptible to landslide hazards at 42.364% (moderate to very high) countrywide (Table 5).

Currently, the increase of population has created serious pressure on land, whereby some families continue to settle in unsafe zones, which increases the number of people impacted by natural disasters including landslides. In other words, exposure is always increasing. In addition, underdeveloped countries are generally unable to cope with natural disaster due to their high vulnerability and low capacity to build resilience [66]. For disaster management framework, a disaster risk is a product of a hazard and vulnerability [67]. For Rwanda, some steep slopes areas that used to be open lands in the past are now covered by family houses; forests have been completely cleared in some places. All of this was mainly caused by the mass influx of returnees after the 1994 war and genocide against Tutsis that devastated Rwanda.

In line with population exposure in Rwanda, 5,828,731 (49.35%) of the total population live in areas categorized as moderate to very high susceptible, with the Western Province (15.2%) the peak, followed by the Southern Province (10.2%). The Eastern Province has the highest population living in the categories of very low landslide susceptibility (6.9%) comparing to the total population of Rwanda. Overall, the eastern can be categorized as a stable zone to landslide disasters. Even though various areas of the country are exposed to landslide hazards, their levels of vulnerabilities are not the same, and each area presents its own particularities. This requires further detailed studies to explain the phenomenon.

Table 5 highlights the level of population exposure to landslide hazards and disasters per province, under different categories of susceptibility. Provinces and districts are not exposed at the same level, and this is subject to several factors including the population density, topographic nature, total surface area, number of total inhabitants available in the provinces and districts, etc.

## 4. Discussion

The landslide hazards have been studied by applying several methodologies and new approaches have been availed to overcome encountered challenges. The Spatial Multi-Criteria Evaluation model (SMCE), therefore, has been confirmed as a good tool in predicting slope stability for large scale areas, prone to landslide disasters [36]. Table 5 and Table 6 display visible information on landslide susceptibility for the entire territory of Rwanda. Furthermore, a landslide susceptibility map divides a given location under various classes that range from stable to landslide hazards [68] and this portrays the spatial distribution of the actual and potential slope failures [69]. This is what Rwanda was lacking to minimize all related shocks by mainstreaming landslide mitigation into national, local and sectoral programs and agenda. It has been revealed that 50% of Rwanda is classified as low susceptible against 7.6% which is under very low susceptibility category (stable zone). Provinces are also exposed to landslides at different levels for several reasons including physical aspects in relation with causal factors considered by this study.

The susceptibility map can easily provide a quick glance at the tendency of mitigation and prevention initiatives to be adopted for disaster management. The relocation of families from landslide high risk zones to safer areas can be considered base on what this study revealed in terms of landslides susceptibility. Thus, safe areas have been disclosed by the produced susceptibility maps. The moderate category of landslide susceptibility (23.64%) is scattered in almost all parts of Rwanda, and this is also considered as an area where stable zones can be found in terms of reducing the likelihood of being affected by landslide incidents. This category counts 2,977,695 people, which represents 25.2% of the total population of Rwanda. This is therefore a high population, which is mostly composed of poor rural families with high vulnerability and low capacity to contain any landslides effects.

**Figure 10:**
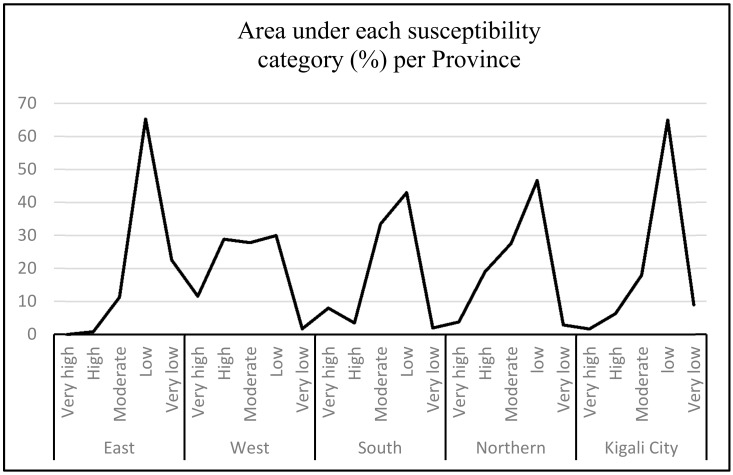
Area under susceptibility category by Province.

The total area of Rwanda is exposed to landslide hazards and disasters at different levels in terms of percentage. The peak for the very high susceptibility category becomes the western province, while, inversely, the eastern province becomes the least exposed to very high landslides (Figure 10).

Generally, the study revealed that almost all provinces of the study area, have zones categorized as moderately susceptible, and these areas also need more serious attention to minimize landslide disaster frequency and intensity. A landslide hazard becomes a disaster in terms of caused impacts and effects [70]; appropriate efforts must be generally put into preventing hazards from turning into disasters. The United Nations International Strategy for Disaster Reduction (UNISDR) confirmed that all disasters are preventable [2]. This should serve as a guide for countries to put into place strong measures, especially for rapid onset disasters.

Steep slopes areas, high elevated zones and high precipitation regions become riskier in terms of landslides and this is aggravated, in some cases, by anthropogenic activities including poor agriculture practices, unplanned settlements, poor construction materials, lack of rain water drainage in place and others. Given that 58.30% of Rwandan land is classified as cropland (Figure 6), landslides affect extensive areas with crops and this obstructs development initiatives and again disturbs food security on many occasions. Hectares of crops are continually washed by landslide hazards. In addition, the disaster risk reduction has a continuum of steps to be accomplished by all involved actors [52], but, when it comes to landslide disasters, the susceptibility assessment and mapping becomes the cornerstone in securing lives and properties. While the susceptibility map could inform urban and land use planning, it can also be an important source of information for different users in general, and particularly risk managers [17].

Moreover, the good modeling of any landslide hazard must be preceded by a generation of inventory maps [71] since it allows developing knowledge and information about landslide frequency and all other necessary information required by researchers as well as potential causal factors. This is what the current study followed as a guiding principle for the whole process towards the production of the national scale landslide susceptibility maps of Rwanda. The Spatial Multi-Criteria approach helped the researchers generate a good landslide susceptibility map with a good scale of 1:1,000,000. It is a national scale susceptibility map that can be used to locate areas for regional mitigation and inform national periodic disaster risk management activities. From this national susceptibility map, it will be possible to deduce susceptibility information at different administrative levels (national, municipal and provincial) and to obtain local susceptibility maps at a high scale of 1:25,000, hence guide the urban and land use planning processes.

**Figure 11:**
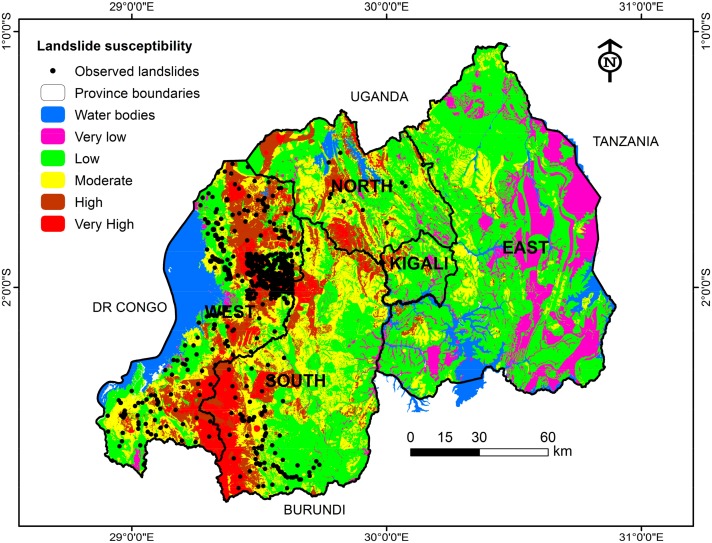
Landslide susceptibility map with past observed landslides.

There are many different ways to come up with landslide susceptibility maps but all of them are guided by the scope, objectives, availability of data, scale of the study area, available resources, etc. [6]. To evaluate the applied landslide susceptibility assessment model, a landslide inventory dataset was used for comparison and validation. The comparison helps to check whether the model produced the reality on the ground or not. This includes 980 past locations of Rwanda affected by landslides which validated the outcome of the SMCE approach (Figure 11).

The comparison showed that about 49.5% of the known landslides lie within the very high susceptibility category, 28% in the category of high susceptibility, approximately 17.8% of known landslides fell into the moderate susceptibility category, and about 4.7% of landslides lie within the low susceptible class. Furthermore, the category of very low susceptibility, which can be considered as non-susceptible or stable zone, dominates entirely the eastern part of Rwanda, and no past landslide location was encountered in this category. This confirms that the produced susceptibility map reflects the ground reality in terms of landslide disasters and hazards. More attention should be put on the categories of high to very high by putting in place appropriate measures to build community disaster resilience, and enhance disaster preparedness measures.

Different parts of Rwanda are known to be affected by landslides mainly from March to May and from October to December due to high to extreme precipitation [37,38,52]. With reference to the altitude and slope of Rwanda, the eastern part is largely dominated by low lands and this expresses its low risk and low susceptibility rank to landslide hazards. Besides, it is a region known to have shortage of rains from many years.

It would therefore be believed that Rwanda is a landslide prone country but this can be controlled by putting in place strong and appropriate measures. All disasters are manageable and all risks are reducible and transferrable [67]. This is justified by the fact that 5.8 million (49.3%) of Rwandans live in the susceptibility categories of moderate to very high susceptibility. Disaster risk management is everybody’s responsibility, and this landslide problem must be looked at in a wider and broader manner to involve all stakeholders to save lives and protect properties [67,70]. For this, landslide mitigation has to be considered as a cross cutting area in Rwanda. By adopting this, it would be very possible control and avert landslide disasters with all its associated adverse impacts and effects. Rwanda, fondly referred to as “the land of a thousand hills” [33], would become a landslide free haven for all its citizens. Scientific findings and good political willingness would devise the paradigm shift from reactive to proactive in relation to landslide disasters. Landslide is a very big killer when it comes to poor rural and vulnerable families unable to absorb and withstand its bad impacts and effects.

## 5. Conclusions

In this research, the authors used the semi-quantitative approach (SMCE-GIS) because of the study area context, objectives, the entire Rwanda aspects and the historical research background and nature. In this study, SMCE model was used to study and map landslide susceptibility in Rwanda. By using eight causal factors, a national landslide susceptibility map was generated with five different categories from very stable to unstable. The generated susceptibility map has a national scale of 1:1,000,000. The validation was done by applying 980 past landslide locations nationwide and the results were found very satisfactory. This confirmed unreservedly that Spatial Multi-Criteria Evaluation model (SMCE) in GIS-ILWIS can predict landslide hazards in Rwanda. It is thus important to mention that, due to the dynamic nature of the land cover/use, precipitation patterns and various anthropogenic activities or any other causal factors, the final susceptibility maps are not static over time. The study has confirmed that landslide hazard is a reality for Rwanda, especially in the western, southern and northern parts. It is within these areas where many losses induced by landslides are repeatedly encountered.

The results demonstrated how landslide susceptibility is spatially distributed, and, as validated by the past landslide locations data, 5.012% of Rwanda was classified as very high susceptible to landslides. This extends to all parts of Rwanda, except the eastern part, which is entirely flat. In addition, the produced susceptibility map will serve in different ways to inform decision making for disaster risk reduction. Additionally, the results of the current study will be useful for infrastructure development, urban planning at district and sector levels, guide relocation of families from high risk zones, awareness creation and education, revision of contingency plans, mainstreaming of disaster risk reduction in other development programs, and decentralization of disaster management funds to the district level. Additionally, to reduce existing uncertainties and limitations, future quantitative studies on landslide susceptibility in Rwanda are recommended with a focus on establishing and maintaining national landslide inventories. This would therefore allow the application of data driven approaches such as statistical and physically-based models. It would then also be reasonable to consider more causal factors to conduct further studies on landslide susceptibility in the study area and obtain strong solutions to the current threat.

## Figures and Tables

**Table 1:** Latest main disasters caused by landslides s in Rwanda.

Time	Place/Venue	Deaths and Injuries	Other Damages
April 2017	Muhanga/South	6 deaths and 27 injured	55 houses destroyed
May 2016	Gakenke/North	35 people killed and 26 injured	67 roads and 29 bridges
May 2016	Muhanga/South	8 people killed and 13 injured	5 roads damaged
May 2016	Rubavu/West	4 people killed and 5 injured	2 bridges destroyed
May 2016	Ngororero/West	13 deaths and 27 injuries	4 classrooms destroyed
April 2015	Ngororero/West	10 deaths and 13 injuries	24 houses destroyed
March 2013	Nyarugenge/Kigali	4 people killed and 3 injured	87 houses destroyed
April 2013	Gasabo/Kigali	3 people killed and 7 injured	56 houses destroyed
May 2013	Rulindo/North	12 people killed and 7 injured	79 houses destroyed
May 2013	Rutsiro/West	5 people killed and 2 injured	22 houses destroyed
May 2011	Nyabihu/West	14 people killed and 11 injured	300 houses destroyed

**Table 2:** Summary of used datasets. The table illustrates major datasets used to model the landslide susceptibility in Rwanda.

Factor/Datasets	Class/Category	Source	Accuracy/Description
Landslide inventory	980 Landslide locations identified	- Field data in Rwanda (January–September 2017) - Secondary data source - Aerial photographs	1:250,000 Scale
DEM (m)	920–1537 m 1537–1831 m 1831–2196 m 2196–2812 m 2812–4494 m	ASTER: United States Geological Surveys	30 × 30 m
Rwanda Land cover/use 2016	Built-Up Cropland Forestland Grassland Water bodies Wetland	Landsat-8 images delivered by the United States Geological Survey (US GS)	30 × 30 m
Lithological features	Basalt Basic igneous rock Colluvial Fluvial Granite Organic Quartzite Schist Volcanic ash	Geological map of Rwanda Rwanda Natural Resources Authority	1:100,000 scale
Soil datasets	[Sandylay-Loam] [Sandy-Clay] [Loam] [Clay-Loam] [Clay]	Rwanda Agriculture Board/Ministry of Agriculture	1:100,000 scale
Road network datasets (distance from main roads in m)	>100 m 100–300 m 300–500 m 500–100 m >1000 m	Rwanda Transport Development Agency/Ministry of Infrastructure	1.50,000 Scale
Precipitation datasets monthly and annual mean/mm	>100 mm (100–120) (120–140) (140–160) (>160)	Rwanda Meteorological Agency [19] and Climate Hazards Group (CHG), Climate Hazards Groups InfraRed Precipitation with Station data (CHIRPS) [54,55]	Monthly and annual mean for 44 years (1972–2016)
Population datasets 11,809,295 inhabitants	Rwanda Population up to June 2017 per Province: East: 2,242,132 West: 2,841,196 North: 1,977,076 South: 3,000,391 City of Kigali: 1,748,500	Rwanda National Institute of Statistics [56] administrative divisions of countries-Statoids [58]	Population database up to June 2017 (by Districts/Province)
Country, Province and Districts Boundaries/Rwanda	Boundaries/Shapefiles	Rwanda Lands and Mapping Departments [63]	Updated boundary shape files of 2014

**Table 3:** Multi-criteria decision matrix in Geographical Information System (GIS)-Integrated Land and Water Information System (ILWIS)-Spatial Multi-Criteria Evaluation (SMCE).

	Y_1 (W1W2W3 ….. Wn)_	Y_2_	Y_3_	…	Y_n_
X_1_	X_11_	X_12_	X_13_	…	X_1n_
X_2_	X_21_	X_22_	X_23_	…	X_2n_
.	.	.	.	…	.
X_m_	X_m1_	X_m2_	X_m3_	…	X_mn_

**Table 4:** Generated weights for causal factors in GIS-LWIS_SMCE. This table highlights weights generated by the model basing on past landslides characteristics.

No.	Causal Factor	Generated and Assigned Weight by the Model	Percentage %
1	Lithology	0.10	10
2	Soil Texture	0.14	14
3	Rainfall	0.2	20
4	Slope	0.2	20
5	Altitude	0.15	15
6	Land cover	0.09	9
7	Soil Depth	0.07	7
8	Distance to Main Roads	0.05	5
	Total	1.00	100

**Table 5:** Rwanda Population exposure within each susceptibility category per province.

Province	Susceptibility Class	Area (%)	Exposure of Local Population
East	Very high	0	0
	High	0.85	196,122
	Moderate	11.28	511,925
	Low	65.3	718,614
	Very low	22.57	815,471
West	Very high	11.59	416,235
	High	28.88	648,582
	Moderate	27.82	734,875
	Low	30	728,390
	Very low	1.71	313,114
South	Very high	8	209,113
	High	3.5	108,378
	Moderate	33.5	881,209
	Low	43	1,005,764
	Very low	2	795,927
Northern	Very high	3.8	224,359
	High	19	400,764
	Moderate	27.6	418,418
	low	46.7	625,330
	Very low	2.9	308,205
Kigali City	Very high	1.67	21,020
	High	6.33	626,463
	Moderate	18	431,268
	low	65	242,904
	Very low	9	426,845
Total	-	100	11,809,295

**Table 6:** Past landslides records compared with produced classes.

No.	Past Landslides Records	Susceptible Areas	Area under Category (%)	Comparison of Past landslides with Landslide Coverage (%)
1	0	Very low	7.636	0
2	46	Low	50	4.7
3	175	Moderate	23.64	17.8
4	274	High	13.712	28
5	485	Very High	5.012	49.5
Total	980		100	100

## Abbreviations

ILWIS	Integrated Land and Water Information System
MIDIMAR	Ministry of Disaster Management and Refugees
MINALOC	Ministry of Local Government
MINAGRI	Ministry of Agriculture
RNRA	Rwanda Natural Resources Authority
UCAS	University of Chinese Academy of Sciences
XIEG	Xinjiang Institute of Ecology and Geography
USD	U.S. Dollar
